# (1*R*,3*S*)-*N*-Benzhydryl-2-benzyl-6,7-dimeth­oxy-1-phenyl-1,2,3,4-tetra­hydro­isoquinoline-3-carbothio­amide

**DOI:** 10.1107/S1600536811049324

**Published:** 2011-11-25

**Authors:** Tricia Naicker, Thavendran Govender, Hendrick. G. Kruger, Glenn E. M. Maguire

**Affiliations:** a School of Pharmacy and Pharmacology, University of KwaZulu-Natal, Durban 4000, South Africa; b School of Chemistry, University of KwaZulu-Natal, Durban 4000, South Africa

## Abstract

The title compound, C_38_H_36_N_2_O_2_S, has a heterocyclic ring that assumes a half-chair conformation. The phenyl rings of neighbouring mol­ecules align forming alternating chains parallel to [100] within the crystal packing. The absolute stereochemistry of the crystal was confirmed to be *R*,*S* at the 1- and 3-positions, respectively, by proton NMR spectroscopy. A single intra­molecular N—H⋯N hydrogen bond is observed.

## Related literature

For background to chiral organocatalysts bearing a tetra­hydro­isoquinoline framework and for related structures, see: Naicker *et al.* (2010 ▶, 2011**a* ▶,b* ▶).
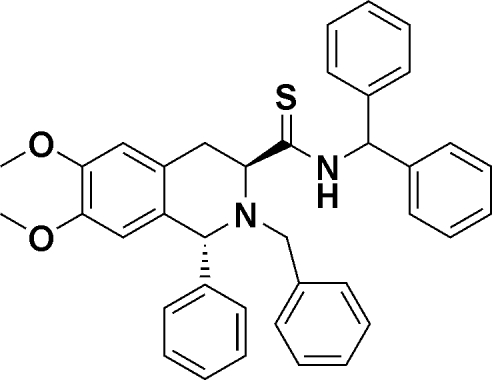


## Experimental

### 

#### Crystal data

C_38_H_36_N_2_O_2_S
*M* *_r_* = 584.75
Orthorhombic, 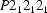

*a* = 9.0463 (1) Å
*b* = 17.6687 (2) Å
*c* = 19.6178 (2) Å
*V* = 3135.64 (6) Å^3^
*Z* = 4
Mo *K*α radiation
μ = 0.14 mm^−1^
*T* = 173 K
0.34 × 0.32 × 0.30 mm

#### Data collection

Nonius KappaCCD diffractometer
7464 measured reflections
7464 independent reflections
6545 reflections with *I* > 2σ(*I*)
*R*_int_ = 0.013

#### Refinement

*R*[*F* ^2^ > 2σ(*F* ^2^)] = 0.033
*wR*(*F* ^2^) = 0.090
*S* = 1.06
7464 reflections
394 parameters
H atoms treated by a mixture of independent and constrained refinement
Δρ_max_ = 0.19 e Å^−3^
Δρ_min_ = −0.25 e Å^−3^
Absolute structure: Flack (1983 ▶), 3271 Friedel pairs
Flack parameter: −0.07 (5)

### 

Data collection: *COLLECT* (Nonius, 2000 ▶); cell refinement: *DENZO-SMN* (Otwinowski & Minor, 1997 ▶); data reduction: *DENZO-SMN*; program(s) used to solve structure: *SHELXS97* (Sheldrick, 2008 ▶); program(s) used to refine structure: *SHELXL97* (Sheldrick, 2008 ▶); molecular graphics: *OLEX2* (Dolomov *et al.*, 2009) ▶; software used to prepare material for publication: *SHELXL97*.

## Supplementary Material

Crystal structure: contains datablock(s) I, global. DOI: 10.1107/S1600536811049324/hg5134sup1.cif

Structure factors: contains datablock(s) I. DOI: 10.1107/S1600536811049324/hg5134Isup2.hkl

Supplementary material file. DOI: 10.1107/S1600536811049324/hg5134Isup3.cml

Additional supplementary materials:  crystallographic information; 3D view; checkCIF report

## Figures and Tables

**Table 1:** Hydrogen-bond geometry (Å, °)

*D*—H⋯*A*	*D*—H	H⋯*A*	*D*⋯*A*	*D*—H⋯*A*
N2—H1*N*⋯N1	0.903 (17)	2.139 (16)	2.6548 (15)	115.4 (12)

**Fig. 1.**
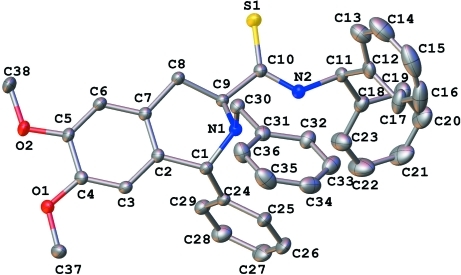
The molecular structure of the title compound with the atom numbering scheme. Displacement ellipsoids are drawn at the 40% probability level. Hydrogen atoms have been omitted for clarity.

**Fig. 2.**
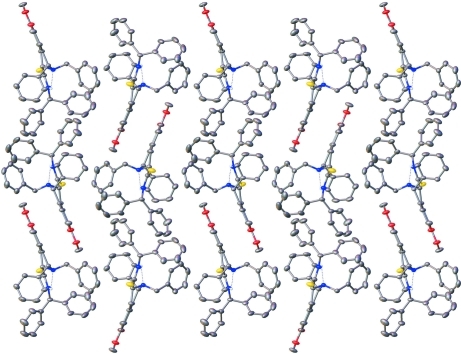
A partial projection of the title compound, viewed along the [100] plane.

## supplementary crystallographic information

### Comment

Chiral organocatalysts bearing a tetrahydroisoquinoline (TIQ) framework have proven to be very successful by our research group (Naicker *et al.*, 2010 and 2011*a*). The title compound (Fig. 1) is a precursor in the synthesis of these novel chiral organocatalysts. The crystal structure contains a thioamide moiety at the C10 position making it the first example in this class to be reported.

The absolute stereochemistry of the molecule was confirmed to be *R,S* at C1 and C9 positions respectively by proton NMR spectroscopy.

The *N*-containing six membered ring assumes a half chair conformation [Q=0.5212 (12) Å, θ= 50.52 (14)° and φ=325.8 (18)°] similar to an analogous structure which has a methyl ester at the C10 position (Naicker *et al.*, 2011*b*). This heterocyclic ring shape affects the position of the thioamide moiety relative to the phenyl ring at the C1 position. The torsion angle for C1—N1—C9—C10 is -157.6 (1)°. Also, in the analogous structure the torsion angle between C8—N1—C9—C10 is 44.1 (2)° while in the title structure this angle is -18.3 (2)°. This is probably due to the C═S bond which adopts a more planar orientation relative to the TIQ backbone as compared to the C═O bond orientation previously reported in this family of molecules (Naicker *et al.*, 2011*b*). In addition, the *N*-benzyl and phenyl ring at C1 exist in a *trans* orientation along the N1—C9 bond with a dihedral angle of -153.3 (1)°.

The title compound contains four phenyl rings however, no intermolecular C—H···π or π···π interactions are evident. A single intramolecular hydrogen bond between atoms N2—H1N···N1 can be observed. The molecules within the crystal structure line up such that the phenyl rings face each other, this forms alternating chains parallel to the [100] plane (Fig. 2).

### Experimental

To a solution of (1*R*,3*S*)-*N*-benzhydryl-2-benzyl-6,7-dimethoxy-1-phenyl-1,2,3,4-tetrahydroisoquinoline-3-carboxamide (0.1 g, 0.02 mmol) in dry THF (20 ml), Lawssons reagent (0.06 g, 0.15 mmol) was added. The mixture was allowed to stir at 50 °C for 16 h under a nitrogen atmosphere. Thereafter the solvent was evaporated *in vacuo* and the residue purified using silica column chromatography (hexane: ethyl acetate, 50:50, *R*_f_ = 0.8) to yield the pure product (0.1 g, 90%) as a yellow solid. *M*.p. = 458 K

Recrystallization from ethyl acetate at room temperature afforded crystals suitable for X-ray analysis.

### Refinement

All non-hydrogen atoms were refined anisotropically. All hydrogen atoms could be found in the difference electron density maps. H1N was thus positioned and refined freely with independent isotropic temperature factors. The other hydrogen atoms were placed with idealized positions and refined as riding on their parents atoms with *U*_iso_ = 1.2 or 1.5 *x U*_eq_ (C).

### Crystal data

**Table:**

C_38_H_36_N_2_O_2_S	*D*_x_ = 1.239 Mg m^−^^3^
*M**_r_* = 584.75	Melting point: 458 K
Orthorhombic, *P*2_1_2_1_2_1_	Mo *K*α radiation, λ = 0.71073 Å
Hall symbol: P 2ac 2ab	Cell parameters from 7464 reflections
*a* = 9.0463 (1) Å	θ = 2.4–27.9°
*b* = 17.6687 (2) Å	µ = 0.14 mm^−^^1^
*c* = 19.6178 (2) Å	*T* = 173 K
*V* = 3135.64 (6) Å^3^	Block, colourless
*Z* = 4	0.34 × 0.32 × 0.30 mm
*F*(000) = 1240	

### Data collection

**Table:**

Nonius KappaCCD diffractometer	6545 reflections with *I* > 2σ(*I*)
Radiation source: fine-focus sealed tube	*R*_int_ = 0.013
graphite	θ_max_ = 27.9°, θ_min_ = 2.4°
1.2° φ scans and ω scans	*h* = −11→11
7464 measured reflections	*k* = −23→23
7464 independent reflections	*l* = −25→25

### Refinement

**Table:**

Refinement on *F*^2^	Secondary atom site location: difference Fourier map
Least-squares matrix: full	Hydrogen site location: inferred from neighbouring sites
*R*[*F*^2^ > 2σ(*F*^2^)] = 0.033	H atoms treated by a mixture of independent and constrained refinement
*wR*(*F*^2^) = 0.090	*w* = 1/[σ^2^(*F*_o_^2^) + (0.0573*P*)^2^ + 0.1291*P*] where *P* = (*F*_o_^2^ + 2*F*_c_^2^)/3
*S* = 1.06	(Δ/σ)_max_ < 0.001
7464 reflections	Δρ_max_ = 0.19 e Å^−^^3^
394 parameters	Δρ_min_ = −0.25 e Å^−^^3^
0 restraints	Absolute structure: Flack (1983), 3271 Friedel pairs
Primary atom site location: structure-invariant direct methods	Flack parameter: −0.07 (5)

### Special details

**Table:**

Geometry. All e.s.d.'s (except the e.s.d. in the dihedral angle between two l.s. planes) are estimated using the full covariance matrix. The cell e.s.d.'s are taken into account individually in the estimation of e.s.d.'s in distances, angles and torsion angles; correlations between e.s.d.'s in cell parameters are only used when they are defined by crystal symmetry. An approximate (isotropic) treatment of cell e.s.d.'s is used for estimating e.s.d.'s involving l.s. planes.
Refinement. Refinement of *F*^2^ against ALL reflections. The weighted *R*-factor *wR* and goodness of fit *S* are based on *F*^2^, conventional *R*-factors *R* are based on *F*, with *F* set to zero for negative *F*^2^. The threshold expression of *F*^2^ > σ(*F*^2^) is used only for calculating *R*-factors(gt) *etc*. and is not relevant to the choice of reflections for refinement. *R*-factors based on *F*^2^ are statistically about twice as large as those based on *F*, and *R*- factors based on ALL data will be even larger.

### Fractional atomic coordinates and isotropic or equivalent isotropic displacement parameters (Å^2^)

**Table:**

	*x*	*y*	*z*	*U*_iso_*/*U*_eq_	
S1	0.56498 (4)	0.31046 (3)	0.215098 (18)	0.04475 (11)	
O1	1.39454 (9)	0.35968 (6)	−0.01266 (5)	0.0361 (2)	
O2	1.15098 (11)	0.39105 (6)	−0.07511 (5)	0.0412 (3)	
N1	0.98038 (11)	0.23680 (6)	0.21258 (5)	0.0254 (2)	
H1N	0.8629 (19)	0.2485 (9)	0.3066 (8)	0.038 (4)*	
N2	0.76795 (13)	0.26373 (7)	0.30274 (5)	0.0300 (2)	
C1	1.12804 (13)	0.26381 (7)	0.19152 (6)	0.0258 (2)	
H1	1.1954	0.2189	0.1909	0.031*	
C2	1.12788 (13)	0.29751 (7)	0.11981 (6)	0.0252 (2)	
C3	1.26341 (13)	0.31006 (7)	0.08659 (6)	0.0258 (2)	
H3	1.3530	0.2963	0.1087	0.031*	
C4	1.26841 (14)	0.34205 (7)	0.02236 (6)	0.0277 (3)	
C5	1.13534 (14)	0.36025 (8)	−0.01141 (6)	0.0295 (3)	
C6	1.00254 (14)	0.34772 (7)	0.02100 (6)	0.0296 (3)	
H6	0.9130	0.3605	−0.0015	0.036*	
C7	0.99719 (13)	0.31627 (7)	0.08690 (6)	0.0263 (2)	
C8	0.84760 (13)	0.30508 (8)	0.12010 (6)	0.0281 (3)	
H8A	0.7988	0.2595	0.1012	0.034*	
H8B	0.7836	0.3494	0.1111	0.034*	
C9	0.87122 (13)	0.29575 (7)	0.19647 (6)	0.0254 (3)	
H9	0.9174	0.3443	0.2118	0.030*	
C10	0.73303 (14)	0.28667 (7)	0.24034 (6)	0.0273 (3)	
C11	0.66652 (15)	0.25452 (8)	0.36016 (6)	0.0307 (3)	
H11	0.5659	0.2705	0.3447	0.037*	
C12	0.66026 (16)	0.17078 (8)	0.37684 (7)	0.0365 (3)	
C13	0.57712 (19)	0.12406 (10)	0.33433 (10)	0.0513 (4)	
H13	0.5187	0.1457	0.2991	0.062*	
C14	0.5791 (2)	0.04658 (12)	0.34297 (13)	0.0717 (6)	
H14	0.5222	0.0151	0.3138	0.086*	
C15	0.6627 (3)	0.01513 (11)	0.39351 (13)	0.0783 (7)	
H15	0.6639	−0.0383	0.3991	0.094*	
C16	0.7452 (3)	0.05997 (12)	0.43635 (10)	0.0724 (7)	
H16	0.8025	0.0375	0.4715	0.087*	
C17	0.7449 (2)	0.13879 (10)	0.42834 (8)	0.0519 (4)	
H17	0.8020	0.1699	0.4578	0.062*	
C18	0.71133 (15)	0.30534 (8)	0.41918 (7)	0.0355 (3)	
C19	0.62417 (18)	0.30449 (10)	0.47767 (7)	0.0461 (4)	
H19	0.5398	0.2725	0.4797	0.055*	
C20	0.6594 (2)	0.34981 (12)	0.53304 (9)	0.0596 (5)	
H20	0.5989	0.3490	0.5726	0.071*	
C21	0.7820 (2)	0.39607 (13)	0.53074 (10)	0.0679 (6)	
H21	0.8059	0.4270	0.5688	0.081*	
C22	0.8697 (2)	0.39761 (14)	0.47359 (11)	0.0721 (6)	
H22	0.9541	0.4296	0.4720	0.087*	
C23	0.8343 (2)	0.35184 (11)	0.41767 (9)	0.0533 (4)	
H23	0.8953	0.3527	0.3783	0.064*	
C24	1.18222 (13)	0.31673 (8)	0.24787 (6)	0.0274 (3)	
C25	1.21466 (16)	0.28510 (9)	0.31128 (7)	0.0377 (3)	
H25	1.2090	0.2318	0.3171	0.045*	
C26	1.25474 (18)	0.32987 (11)	0.36556 (7)	0.0494 (4)	
H26	1.2770	0.3073	0.4083	0.059*	
C27	1.2627 (2)	0.40751 (11)	0.35813 (8)	0.0525 (4)	
H27	1.2896	0.4384	0.3957	0.063*	
C28	1.23137 (18)	0.43995 (10)	0.29567 (8)	0.0461 (4)	
H28	1.2370	0.4933	0.2902	0.055*	
C29	1.19146 (15)	0.39432 (8)	0.24068 (7)	0.0347 (3)	
H29	1.1704	0.4169	0.1978	0.042*	
C30	0.94220 (15)	0.16198 (7)	0.18354 (6)	0.0311 (3)	
H30A	0.9619	0.1621	0.1339	0.037*	
H30B	0.8356	0.1519	0.1905	0.037*	
C31	1.03199 (15)	0.10040 (7)	0.21717 (7)	0.0313 (3)	
C32	1.00795 (17)	0.08224 (8)	0.28514 (7)	0.0369 (3)	
H32	0.9328	0.1078	0.3099	0.044*	
C33	1.09188 (18)	0.02740 (9)	0.31743 (9)	0.0449 (4)	
H33	1.0739	0.0154	0.3639	0.054*	
C34	1.20184 (17)	−0.00989 (8)	0.28199 (10)	0.0483 (4)	
H34	1.2612	−0.0466	0.3044	0.058*	
C35	1.22548 (19)	0.00598 (9)	0.21423 (10)	0.0535 (4)	
H35	1.2999	−0.0203	0.1896	0.064*	
C36	1.13978 (18)	0.06090 (9)	0.18180 (9)	0.0442 (4)	
H36	1.1556	0.0713	0.1349	0.053*	
C37	1.53137 (14)	0.34969 (9)	0.02154 (7)	0.0368 (3)	
H37A	1.5315	0.3794	0.0637	0.055*	
H37B	1.6122	0.3667	−0.0080	0.055*	
H37C	1.5449	0.2960	0.0325	0.055*	
C38	1.01921 (18)	0.40522 (12)	−0.11254 (8)	0.0523 (4)	
H38A	0.9625	0.3582	−0.1168	0.078*	
H38B	1.0446	0.4240	−0.1580	0.078*	
H38C	0.9597	0.4432	−0.0886	0.078*	

### Atomic displacement parameters (Å^2^)

**Table:**

	*U*^11^	*U*^22^	*U*^33^	*U*^12^	*U*^13^	*U*^23^
S1	0.02261 (15)	0.0813 (3)	0.03029 (17)	0.00153 (17)	−0.00103 (14)	0.01267 (18)
O1	0.0218 (4)	0.0640 (6)	0.0226 (4)	−0.0016 (4)	0.0025 (3)	0.0056 (5)
O2	0.0294 (5)	0.0712 (7)	0.0230 (5)	−0.0013 (5)	−0.0009 (4)	0.0162 (5)
N1	0.0222 (5)	0.0317 (5)	0.0222 (5)	0.0003 (4)	0.0004 (4)	0.0019 (4)
N2	0.0229 (5)	0.0445 (6)	0.0227 (5)	0.0020 (5)	0.0021 (4)	0.0030 (4)
C1	0.0216 (6)	0.0343 (6)	0.0215 (6)	0.0009 (5)	−0.0017 (5)	0.0020 (5)
C2	0.0250 (6)	0.0323 (6)	0.0183 (5)	0.0005 (5)	0.0010 (5)	−0.0018 (5)
C3	0.0208 (5)	0.0355 (6)	0.0212 (5)	0.0016 (5)	−0.0012 (5)	−0.0014 (5)
C4	0.0243 (6)	0.0380 (6)	0.0208 (6)	−0.0013 (5)	0.0024 (5)	−0.0020 (5)
C5	0.0281 (6)	0.0423 (7)	0.0180 (5)	−0.0022 (5)	−0.0009 (5)	0.0035 (5)
C6	0.0243 (6)	0.0422 (7)	0.0224 (6)	0.0009 (5)	−0.0038 (5)	0.0040 (5)
C7	0.0231 (6)	0.0353 (6)	0.0204 (5)	−0.0001 (5)	0.0004 (5)	0.0007 (5)
C8	0.0219 (6)	0.0407 (7)	0.0216 (6)	0.0018 (5)	−0.0007 (5)	0.0039 (5)
C9	0.0200 (5)	0.0355 (6)	0.0207 (6)	−0.0001 (5)	−0.0004 (4)	0.0021 (5)
C10	0.0238 (6)	0.0351 (6)	0.0230 (6)	−0.0022 (5)	0.0002 (5)	0.0013 (5)
C11	0.0255 (6)	0.0458 (8)	0.0206 (6)	0.0015 (6)	0.0038 (5)	0.0012 (5)
C12	0.0341 (7)	0.0478 (8)	0.0275 (6)	0.0034 (6)	0.0126 (6)	0.0038 (6)
C13	0.0409 (9)	0.0509 (9)	0.0622 (11)	−0.0062 (7)	0.0103 (8)	−0.0039 (8)
C14	0.0596 (12)	0.0533 (11)	0.1023 (18)	−0.0122 (9)	0.0277 (13)	−0.0074 (12)
C15	0.1006 (17)	0.0443 (10)	0.0899 (16)	0.0067 (11)	0.0567 (15)	0.0114 (11)
C16	0.1033 (17)	0.0658 (12)	0.0480 (10)	0.0370 (13)	0.0324 (12)	0.0247 (10)
C17	0.0691 (11)	0.0576 (9)	0.0289 (7)	0.0223 (9)	0.0124 (7)	0.0087 (7)
C18	0.0349 (7)	0.0471 (8)	0.0244 (6)	0.0062 (6)	−0.0002 (5)	−0.0011 (6)
C19	0.0486 (9)	0.0604 (9)	0.0294 (7)	0.0029 (8)	0.0071 (7)	−0.0055 (7)
C20	0.0639 (11)	0.0822 (13)	0.0326 (8)	0.0110 (10)	0.0034 (8)	−0.0154 (8)
C21	0.0647 (12)	0.0924 (14)	0.0466 (10)	0.0085 (11)	−0.0098 (9)	−0.0324 (10)
C22	0.0525 (11)	0.0978 (15)	0.0660 (13)	−0.0125 (10)	−0.0053 (10)	−0.0334 (12)
C23	0.0420 (9)	0.0742 (11)	0.0438 (9)	−0.0045 (8)	0.0056 (7)	−0.0170 (8)
C24	0.0192 (5)	0.0433 (7)	0.0198 (6)	0.0003 (5)	0.0000 (4)	−0.0005 (5)
C25	0.0329 (7)	0.0549 (8)	0.0254 (6)	−0.0070 (6)	−0.0057 (6)	0.0076 (6)
C26	0.0445 (9)	0.0813 (12)	0.0225 (6)	−0.0125 (8)	−0.0070 (6)	0.0035 (7)
C27	0.0495 (9)	0.0759 (12)	0.0322 (8)	−0.0116 (9)	−0.0053 (7)	−0.0157 (8)
C28	0.0455 (8)	0.0502 (8)	0.0427 (9)	−0.0019 (7)	−0.0066 (7)	−0.0112 (7)
C29	0.0319 (7)	0.0438 (7)	0.0284 (7)	0.0000 (6)	−0.0050 (5)	−0.0002 (6)
C30	0.0314 (6)	0.0367 (7)	0.0252 (6)	−0.0026 (5)	−0.0017 (5)	−0.0004 (5)
C31	0.0324 (7)	0.0307 (6)	0.0307 (6)	−0.0050 (5)	−0.0009 (5)	−0.0019 (5)
C32	0.0453 (8)	0.0353 (7)	0.0300 (7)	0.0008 (6)	−0.0027 (6)	−0.0012 (6)
C33	0.0562 (10)	0.0364 (7)	0.0421 (8)	−0.0038 (7)	−0.0116 (8)	0.0052 (6)
C34	0.0419 (8)	0.0338 (7)	0.0693 (11)	−0.0036 (6)	−0.0122 (8)	0.0103 (7)
C35	0.0433 (9)	0.0411 (8)	0.0762 (12)	0.0069 (7)	0.0152 (9)	0.0040 (8)
C36	0.0484 (9)	0.0402 (7)	0.0439 (8)	0.0014 (7)	0.0117 (7)	0.0035 (7)
C37	0.0231 (6)	0.0587 (9)	0.0285 (6)	0.0000 (6)	0.0012 (5)	0.0009 (6)
C38	0.0369 (8)	0.0890 (12)	0.0310 (7)	−0.0062 (8)	−0.0069 (6)	0.0259 (8)

### Geometric parameters (Å, °)

**Table:**

S1—C10	1.6532 (13)	C18—C23	1.383 (2)
O1—C4	1.3679 (15)	C18—C19	1.392 (2)
O1—C37	1.4190 (15)	C19—C20	1.387 (2)
O2—C5	1.3704 (15)	C19—H19	0.9500
O2—C38	1.4223 (17)	C20—C21	1.379 (3)
N1—C9	1.4696 (16)	C20—H20	0.9500
N1—C1	1.4773 (15)	C21—C22	1.374 (3)
N1—C30	1.4804 (16)	C21—H21	0.9500
N2—C10	1.3276 (16)	C22—C23	1.400 (3)
N2—C11	1.4621 (16)	C22—H22	0.9500
N2—H1N	0.903 (17)	C23—H23	0.9500
C1—C2	1.5277 (16)	C24—C29	1.381 (2)
C1—C24	1.5286 (17)	C24—C25	1.3950 (18)
C1—H1	1.0000	C25—C26	1.375 (2)
C2—C7	1.3872 (16)	C25—H25	0.9500
C2—C3	1.4061 (17)	C26—C27	1.381 (3)
C3—C4	1.3817 (17)	C26—H26	0.9500
C3—H3	0.9500	C27—C28	1.382 (2)
C4—C5	1.4111 (18)	C27—H27	0.9500
C5—C6	1.3771 (18)	C28—C29	1.394 (2)
C6—C7	1.4080 (17)	C28—H28	0.9500
C6—H6	0.9500	C29—H29	0.9500
C7—C8	1.5148 (17)	C30—C31	1.5096 (19)
C8—C9	1.5223 (16)	C30—H30A	0.9900
C8—H8A	0.9900	C30—H30B	0.9900
C8—H8B	0.9900	C31—C36	1.385 (2)
C9—C10	1.5262 (17)	C31—C32	1.389 (2)
C9—H9	1.0000	C32—C33	1.384 (2)
C11—C12	1.516 (2)	C32—H32	0.9500
C11—C18	1.5202 (19)	C33—C34	1.381 (2)
C11—H11	1.0000	C33—H33	0.9500
C12—C17	1.388 (2)	C34—C35	1.375 (3)
C12—C13	1.394 (2)	C34—H34	0.9500
C13—C14	1.379 (3)	C35—C36	1.396 (2)
C13—H13	0.9500	C35—H35	0.9500
C14—C15	1.365 (4)	C36—H36	0.9500
C14—H14	0.9500	C37—H37A	0.9800
C15—C16	1.375 (4)	C37—H37B	0.9800
C15—H15	0.9500	C37—H37C	0.9800
C16—C17	1.401 (3)	C38—H38A	0.9800
C16—H16	0.9500	C38—H38B	0.9800
C17—H17	0.9500	C38—H38C	0.9800
			
C4—O1—C37	117.50 (10)	C23—C18—C11	123.29 (13)
C5—O2—C38	117.01 (11)	C19—C18—C11	118.05 (13)
C9—N1—C1	108.58 (9)	C20—C19—C18	120.62 (16)
C9—N1—C30	113.17 (10)	C20—C19—H19	119.7
C1—N1—C30	113.06 (10)	C18—C19—H19	119.7
C10—N2—C11	126.52 (11)	C21—C20—C19	120.09 (17)
C10—N2—H1N	113.2 (10)	C21—C20—H20	120.0
C11—N2—H1N	120.0 (10)	C19—C20—H20	120.0
N1—C1—C2	112.49 (9)	C22—C21—C20	120.22 (16)
N1—C1—C24	106.58 (10)	C22—C21—H21	119.9
C2—C1—C24	115.33 (10)	C20—C21—H21	119.9
N1—C1—H1	107.4	C21—C22—C23	119.72 (19)
C2—C1—H1	107.4	C21—C22—H22	120.1
C24—C1—H1	107.4	C23—C22—H22	120.1
C7—C2—C3	119.32 (10)	C18—C23—C22	120.69 (16)
C7—C2—C1	121.50 (10)	C18—C23—H23	119.7
C3—C2—C1	119.17 (11)	C22—C23—H23	119.7
C4—C3—C2	121.04 (11)	C29—C24—C25	118.44 (12)
C4—C3—H3	119.5	C29—C24—C1	123.56 (11)
C2—C3—H3	119.5	C25—C24—C1	117.86 (12)
O1—C4—C3	125.35 (11)	C26—C25—C24	121.03 (15)
O1—C4—C5	115.08 (10)	C26—C25—H25	119.5
C3—C4—C5	119.57 (11)	C24—C25—H25	119.5
O2—C5—C6	125.09 (12)	C25—C26—C27	120.22 (15)
O2—C5—C4	115.50 (11)	C25—C26—H26	119.9
C6—C5—C4	119.40 (11)	C27—C26—H26	119.9
C5—C6—C7	121.15 (11)	C26—C27—C28	119.65 (14)
C5—C6—H6	119.4	C26—C27—H27	120.2
C7—C6—H6	119.4	C28—C27—H27	120.2
C2—C7—C6	119.50 (11)	C27—C28—C29	119.95 (16)
C2—C7—C8	122.00 (10)	C27—C28—H28	120.0
C6—C7—C8	118.49 (11)	C29—C28—H28	120.0
C7—C8—C9	108.18 (10)	C24—C29—C28	120.71 (13)
C7—C8—H8A	110.1	C24—C29—H29	119.6
C9—C8—H8A	110.1	C28—C29—H29	119.6
C7—C8—H8B	110.1	N1—C30—C31	110.48 (10)
C9—C8—H8B	110.1	N1—C30—H30A	109.6
H8A—C8—H8B	108.4	C31—C30—H30A	109.6
N1—C9—C8	112.50 (10)	N1—C30—H30B	109.6
N1—C9—C10	110.78 (10)	C31—C30—H30B	109.6
C8—C9—C10	116.83 (10)	H30A—C30—H30B	108.1
N1—C9—H9	105.2	C36—C31—C32	118.35 (13)
C8—C9—H9	105.2	C36—C31—C30	121.53 (13)
C10—C9—H9	105.2	C32—C31—C30	120.13 (12)
N2—C10—C9	110.92 (10)	C33—C32—C31	121.00 (14)
N2—C10—S1	124.93 (10)	C33—C32—H32	119.5
C9—C10—S1	123.89 (9)	C31—C32—H32	119.5
N2—C11—C12	107.35 (11)	C34—C33—C32	119.92 (16)
N2—C11—C18	110.72 (11)	C34—C33—H33	120.0
C12—C11—C18	114.96 (11)	C32—C33—H33	120.0
N2—C11—H11	107.9	C35—C34—C33	120.09 (15)
C12—C11—H11	107.9	C35—C34—H34	120.0
C18—C11—H11	107.9	C33—C34—H34	120.0
C17—C12—C13	119.47 (15)	C34—C35—C36	119.74 (16)
C17—C12—C11	122.26 (14)	C34—C35—H35	120.1
C13—C12—C11	117.97 (14)	C36—C35—H35	120.1
C14—C13—C12	120.5 (2)	C31—C36—C35	120.86 (15)
C14—C13—H13	119.8	C31—C36—H36	119.6
C12—C13—H13	119.8	C35—C36—H36	119.6
C15—C14—C13	120.0 (2)	O1—C37—H37A	109.5
C15—C14—H14	120.0	O1—C37—H37B	109.5
C13—C14—H14	120.0	H37A—C37—H37B	109.5
C14—C15—C16	120.67 (18)	O1—C37—H37C	109.5
C14—C15—H15	119.7	H37A—C37—H37C	109.5
C16—C15—H15	119.7	H37B—C37—H37C	109.5
C15—C16—C17	120.2 (2)	O2—C38—H38A	109.5
C15—C16—H16	119.9	O2—C38—H38B	109.5
C17—C16—H16	119.9	H38A—C38—H38B	109.5
C12—C17—C16	119.16 (19)	O2—C38—H38C	109.5
C12—C17—H17	120.4	H38A—C38—H38C	109.5
C16—C17—H17	120.4	H38B—C38—H38C	109.5
C23—C18—C19	118.66 (14)		
			
C9—N1—C1—C2	−47.06 (13)	N2—C11—C12—C13	−76.53 (16)
C30—N1—C1—C2	79.40 (12)	C18—C11—C12—C13	159.81 (13)
C9—N1—C1—C24	80.27 (11)	C17—C12—C13—C14	−0.3 (2)
C30—N1—C1—C24	−153.27 (10)	C11—C12—C13—C14	173.60 (15)
N1—C1—C2—C7	14.37 (17)	C12—C13—C14—C15	0.1 (3)
C24—C1—C2—C7	−108.15 (14)	C13—C14—C15—C16	0.2 (3)
N1—C1—C2—C3	−165.91 (11)	C14—C15—C16—C17	−0.4 (3)
C24—C1—C2—C3	71.57 (14)	C13—C12—C17—C16	0.1 (2)
C7—C2—C3—C4	1.25 (18)	C11—C12—C17—C16	−173.46 (15)
C1—C2—C3—C4	−178.48 (11)	C15—C16—C17—C12	0.2 (3)
C37—O1—C4—C3	−5.03 (19)	N2—C11—C18—C23	−1.5 (2)
C37—O1—C4—C5	173.83 (13)	C12—C11—C18—C23	120.33 (16)
C2—C3—C4—O1	176.79 (12)	N2—C11—C18—C19	178.80 (13)
C2—C3—C4—C5	−2.03 (19)	C12—C11—C18—C19	−59.35 (18)
C38—O2—C5—C6	−5.5 (2)	C23—C18—C19—C20	0.5 (2)
C38—O2—C5—C4	175.76 (14)	C11—C18—C19—C20	−179.80 (15)
O1—C4—C5—O2	1.68 (17)	C18—C19—C20—C21	−0.3 (3)
C3—C4—C5—O2	−179.38 (11)	C19—C20—C21—C22	0.1 (3)
O1—C4—C5—C6	−177.17 (12)	C20—C21—C22—C23	−0.1 (3)
C3—C4—C5—C6	1.76 (19)	C19—C18—C23—C22	−0.5 (3)
O2—C5—C6—C7	−179.48 (13)	C11—C18—C23—C22	179.80 (17)
C4—C5—C6—C7	−0.7 (2)	C21—C22—C23—C18	0.3 (3)
C3—C2—C7—C6	−0.20 (18)	N1—C1—C24—C29	−107.96 (13)
C1—C2—C7—C6	179.52 (11)	C2—C1—C24—C29	17.67 (17)
C3—C2—C7—C8	−179.26 (12)	N1—C1—C24—C25	67.68 (14)
C1—C2—C7—C8	0.47 (19)	C2—C1—C24—C25	−166.70 (11)
C5—C6—C7—C2	0.0 (2)	C29—C24—C25—C26	0.1 (2)
C5—C6—C7—C8	179.06 (12)	C1—C24—C25—C26	−175.77 (13)
C2—C7—C8—C9	17.38 (17)	C24—C25—C26—C27	0.4 (2)
C6—C7—C8—C9	−161.69 (11)	C25—C26—C27—C28	−0.5 (3)
C1—N1—C9—C8	69.61 (13)	C26—C27—C28—C29	0.2 (3)
C30—N1—C9—C8	−56.79 (13)	C25—C24—C29—C28	−0.4 (2)
C1—N1—C9—C10	−157.57 (10)	C1—C24—C29—C28	175.21 (13)
C30—N1—C9—C10	76.03 (12)	C27—C28—C29—C24	0.3 (2)
C7—C8—C9—N1	−52.47 (14)	C9—N1—C30—C31	−164.19 (10)
C7—C8—C9—C10	177.76 (10)	C1—N1—C30—C31	71.84 (13)
C11—N2—C10—C9	174.98 (12)	N1—C30—C31—C36	−111.71 (14)
C11—N2—C10—S1	0.6 (2)	N1—C30—C31—C32	68.10 (15)
N1—C9—C10—N2	36.60 (14)	C36—C31—C32—C33	1.6 (2)
C8—C9—C10—N2	167.18 (11)	C30—C31—C32—C33	−178.21 (13)
N1—C9—C10—S1	−148.92 (10)	C31—C32—C33—C34	0.3 (2)
C8—C9—C10—S1	−18.34 (16)	C32—C33—C34—C35	−1.7 (2)
C10—N2—C11—C12	113.87 (15)	C33—C34—C35—C36	1.2 (3)
C10—N2—C11—C18	−119.91 (14)	C32—C31—C36—C35	−2.1 (2)
N2—C11—C12—C17	97.16 (15)	C30—C31—C36—C35	177.71 (14)
C18—C11—C12—C17	−26.50 (19)	C34—C35—C36—C31	0.7 (3)

### Hydrogen-bond geometry (Å, °)

**Table:**

*D*—H···*A*	*D*—H	H···*A*	*D*···*A*	*D*—H···*A*
N2—H1N···N1	0.903 (17)	2.139 (16)	2.6548 (15)	115.4 (12)
